# Effect of Gamma Irradiation and Simulated Physiological Conditions on the Physicochemical Properties of a 3D-Printed βTCP Composite

**DOI:** 10.3390/polym18070817

**Published:** 2026-03-27

**Authors:** Elham Seifi, Sacha Cavelier, Kerr D. G. Samson, Dietmar W. Hutmacher

**Affiliations:** 1 ARC Training Centre for Cell and Tissue Engineering Technologies, Queensland University of Technology, Brisbane 4059, Australia; elham.seifi@hdr.qut.edu.au (E.S.); sacha.cavelier@qut.edu.au (S.C.); 2 School of Mechanical, Medical and Process Engineering, Faculty of Engineering, Queensland University of Technology, Brisbane 4059, Australia; 3 Centre for Biomedical Technologies, Queensland University of Technology, Brisbane 4059, Australia; 4 Max Planck Queensland Centre for the Materials Science of Extracellular Matrices, Queensland University of Technology, Brisbane 4059, Australia; 5 School of Chemistry and Physics, Faculty of Science, Queensland University of Technology, Brisbane 4059, Australia; k3.samson@qut.edu.au; 6 Australian Research Council Training Centre for Multiscale 3D Imaging, Modelling, and Manufacturing, Queensland University of Technology, Brisbane 4059, Australia

**Keywords:** biodegradable composite, hydrated conditions, thermal properties, mechanical properties, hyperelasticity, gamma irradiation

## Abstract

This study investigates the effects of hydration, temperature, and γ-irradiation on the structural, thermal, and mechanical properties of Lactoprene^®^ 7415, a linear block copolymer consisting of 74% lactide, 15% trimethylene carbonate, 11% ε-caprolactone repeating units, and 40 wt% β-TCP/Lactoprene^®^ 7415 composite. Techniques including static and dynamic mechanical testing or differential scanning calorimetry have evidenced structural changes resulting from irradiation- or water-induced crystallinity, crosslinking, chain scission or plasticization. Notably, hydration and physiological temperatures reduced the mechanical properties but conferred hyperelastic characteristics to the polymeric and composite samples. γ-irradiation was detrimental for the mechanical properties, except for those of the pure polymer in dry conditions. Our results evidence a complex interplay between the polymer, particles, temperature, hydration and water. Such observations could have implications in future designs and investigations of composite materials for scaffold-guided bone regeneration (SGBR), such as sterilization processes or minimally invasive surgery.

## 1. Introduction

Linear block copolymers that combine PLA/PLLA (from lactide and L-lactide) with PTMC (from trimethylene carbonate, TMC) and PCL (from ε-caprolactone) were developed to solve a classic biomedical material trade-off more than four decades ago. PLA provides strength but is brittle and can generate acidic degradation products, whereas PTMC and PCL provide flexibility/toughness and slower (often more neutral) degradation profiles. Architectures that place PLA as “hard” end blocks and PTMC/PCL-rich segments as “soft” blocks can behave as biodegradable thermoplastic elastomers (TPEs), i.e., melt-processable materials with elastic recovery [1,2,3,4].

Unfortunately, only recently has a medical-grade version become commercially available to the biomaterial community. One of the defining advantages of Lactoprene^®^ 741, a linear block copolymer consisting of 74% lactide, 15% trimethylene carbonate, and 11% ε-caprolactone repeating units, is its compatibility with conventional thermoplastic processing techniques. The biodegradable polymer can be processed using melt-based additive manufacturing, enabling integration into complex medical device architectures and multi-material assemblies. From a translational standpoint, the processability into filaments suitable for commercial 3D fused deposition modeling printing supports high-throughput, reproducible manufacturing, a key requirement for medical devices [5]. The fabrication technique also allows for mixing 40 wt% of ceramic particles with the polymer without the undesirable effects of high particle contents, such as particle agglomeration. Bioceramic materials are essential in the design of scaffolds for SGBR applications, as they not only improve the mechanical properties, but also biological properties such as osteoconductivity and osteointegration [6,7]. Design strategies usually aim for the highest possible ceramic content without aggregation, but the incorporation of this inorganic phase is also accompanied by mechanistic interactions. For instance, interfacial bonding between the polymer and the ceramic, critical for stress transfer and effective improvement of the mechanical properties, is sensitive to environmental parameters, such as hydration [8]. However, a complete understanding of the interactions between hydration, the ceramic, and the polymer has not been fully elucidated yet. In addition, the impact of sterilization on biomaterial properties must also be considered in the design of biodegradable medical devices.

Gamma irradiation (γ-irradiation) is one of the main techniques, along with ethylene oxide, used to sterilize FDA-approved and CE-marked biodegradable polymeric and composite implants because of its deep penetration into the material, isothermal process, efficiency at high volumes, uniformity, and reliability [9,10,11]. This method, however, has some drawbacks that must be examined from a biomaterial science perspective. Chemical and structural changes can occur in the polymeric phase during γ-irradiation. For instance, chain scission and crosslinking are two dose-dependent competing mechanisms that operate at the molecular scale and affect the mechanical properties [12,13]. Chain scission is the cleavage of C–C bonds along the polymer backbone, and crosslinking is the partial recombination of chain fragments and free radicals [14,15]. The irradiation dose, the atmosphere, and the nature of the polymer are therefore central to determining which mechanism is dominant and how the polymer’s molecular weight and physicochemical properties are modified [14,16,17,18,19,20]. As a result, the effects of radiation on the molecular structure and overall properties of biodegradable polymers and composites appear unpredictable, and the literature provides conflicting predictions for the expected mechanical properties. For instance, Cottam et al. [21] reported that γ-irradiation at a dose of 30.8 kGy significantly increased the yield stress of polycaprolactone (PCL) due to extensive crosslinking in the polymeric structure. On the contrary, studies have reported a detrimental effect of γ-irradiation, even at low doses: a collagen–composite scaffold experienced a decrease in the composite strength after irradiation at 25 kGy [22], PCL exhibited chain scission in the 10–50 kGy dose range [23], the tensile strength of poly(lactic-co-glycolic acid) (PLGA) decreased at irradiation doses as low as 3 kGy [24], and poly-L-lactic acid also had lower mechanical properties after 39 kGy irradiation [25]. Other studies have investigated whether an optimal irradiation dose for the mechanical properties could be identified for each polymer [19]. In the case of aliphatic polyesters, especially homopolymers of PGA, PLA and PCL and copolymers of poly(lactide-*co*-glycolide), it is reported that chain scission is dominant at high doses of *γ*-irradiation [20,25,26] and is counter-balanced by crosslinking at low doses [20,26]. For instance, Narkis et al. [26] reported that the number of scissions equaled and counterbalanced the number of crosslinks in PCL at a dose of 260 kGy. Similarly, Mohammadian-Kohol et al. [27] investigated the impact of γ-irradiation on the physical and mechanical properties of polyvinyl butyral and observed that the threshold was actually 70 kGy for this polymer.

Finally, γ-irradiation can also affect other physicochemical properties. A reduction in crystallinity due to irradiation-induced damage to crystallites has been observed in irradiated PLA and poly-d,l-lactic acid (PDLLA) [28,29]. Conversely, if the polymeric chains become more mobile, they can assemble into more ordered configurations, thereby increasing the number of crystallites but also reducing their size [26,28,29]. A decrease in the melting temperature of PLA was also observed across the 50–1000 kGy range [20], reflecting underlying structural and molecular alterations.

In the context of SGBR, the structural and mechanical effects of γ-irradiation and physiological conditions on biomedical implants must be fully elucidated. However, there are two gaps in the literature: the physicochemical properties of biodegradable polymers and composites have not yet been systematically investigated in physiological conditions, and the effects of γ-irradiation on these materials become challenging to predict when hydration and temperature also contribute to the interplay; this remains under-investigated. In this context, this study aims to examine how γ-irradiation, hydration, and temperature affect the structural, thermal, and mechanical properties of a medical-grade linear block copolymer consisting of 74% lactide, 15% trimethylene carbonate, and 11% ε-caprolactone, either unfilled or filled with a high concentration (40 wt%) of tricalcium phosphate (β-TCP). A series of characterization techniques were used to measure thermal characteristics, compressive properties, degree of crystallinity, molecular weight, and water uptake across different sample groups, γ-irradiated and non-irradiated, under varying hydration states and temperature conditions.

## 2. Materials and Methods

### 2.1. Materials

Cylindrical test specimens, made of pure polymer or composite, were 3D-printed using, respectively, a medical-grade Lactoprene^®^ 7415 mono filament and a medical-grade mono filament composed of 60 wt% Lactoprene^®^ 7415 and 40 wt% β-tricalcium phosphate, supplied by Poly-Med, Inc. (Anderson, SC, USA). Lactoprene^®^ 7415 is a linear block copolymer consisting of 74% lactide, 15% trimethylene carbonate, and 11% ε-caprolactone repeating units. The β-TCP used in the compounding process had an average particle size of 17.3 µm and a β-phase purity more than 95%.

### 2.2. Three-Dimensional Printing

The designs of the cylindrical specimens were exported as stereolithography (STL) files. The files were then imported into the Bambu Studio slicing program, and the G-code toolpath was sent to the 3D printer (Bambu Lab X1, Bambu Lab, Shanghai, China) to print bulk structures. The nozzle and printing platform extrusion temperatures were set to 215 °C and 50 °C, respectively. Print speeds were maintained at 5 mm/s for the first layer and 40 mm/s for internal solid infill. A nozzle diameter of 0.4 mm and a layer height of 0.1 mm were selected to increase the printing resolution of the bulk-like cylinders, ensuring a solid structure with zero porosity. The diameter-to-height ratio of these bulk-like samples was defined in accordance with the ASTM standard for the compressive properties of rigid plastics (ASTM D695-23) [30], with a height of 25.4 mm and a cross-sectional diameter of 12.7 mm. The resulting material was observed under scanning electron microscopy, including under a cross-sectional view after freeze-fracture, particle distribution and surface morphology in a preceding study [5].

The samples were γ-irradiated using a well-established procedure for sterilizing polymer-based medical devices, and each sample received a dose of 25.4 kGy. The procedure was conducted by Steritech, Gold Coast, QLD, Australia in compliance with the ISO 11137 standard [31]. In total, four sample groups were used in the study, as depicted in Table 1.

### 2.3. Differential Scanning Calorimetry

The thermal properties of non-hydrated and hydrated samples from all groups (three samples per group) were determined in triplicate. Hydrated samples were prepared by immersion in phosphate-buffered saline (PBS) at room temperature and at 37 °C. Based on water uptake data previously published [5], immersion times of 30 min, 1 day, and 7 days were selected. The measurements were performed using differential scanning calorimetry (DSC) with a Netzsch DSC 204 F1 Phoenix^®^ (Erich Netzsch GmbH & Co. Holding KG, Selb, Germany) instrument, calibrated with indium. As a reference, an empty 85 μL aluminum pan sealed with a lid was used, while another similar pan was prepared with the sample. Approximately 8 mg of sample was placed in the sample pan, then sealed, pierced, and loaded into the DSC furnace. Both pans were subjected to the same temperature program, consisting of a heat–cool–heat cycle with an initial heating ramp from −40 °C to 250 °C at a rate of 10 °C/min, followed by a cooling stage to −40 °C at 10 °C/min, and finally a repeat of the first heating ramp, all under a nitrogen flow of 20 mL/min. The thermal parameters, such as the glass transition temperature, *T_g_*, the cold crystallization temperature, *T_cc_*, and the melting temperature, *T_m_*, were extracted from the first heating run to reflect the initial crystallinity of the samples, including the effect of 3D printing. The data were evaluated using Netzsch Proteus Thermal Analysis v. 8.0.3. *T_g_* was determined by identifying the midpoint of a stepwise increase in the heat–flow curve, corresponding to a change in the sample’s specific heat capacity. The fraction of freezable water in samples was derived from the area under the heat–flow curve at the melting-temperature peak of water. The crystallinity degree *X_c_* was calculated based on the melting and cold crystallization enthalpy obtained from the first heating cycle, using Equation (1), where ∆*H_m_* is the melting enthalpy of the sample, ∆*H_cc_* is the cold crystallization enthalpy of the sample, ${∆H}_{m,\;PLA}^{0}$ is the standard melting enthalpy of 100% crystalline PLA and is equal to 93 J/g [32], 0.74 relates to the lactide content of the Lactoprene^®^ 7415, and 0.60 relates to the polymer content of the composite, 60 wt% [33].

(1) $$X_{c}=\left(\frac{∆H_{m}-∆H_{cc}}{{\left(0.74\times 0.60\right)\times ∆H}_{m,\;\;\;PLA}^{0}}\right)\times 100$$

The freezable water content was determined by using the enthalpy of water at approximately 0 °C and comparing it with the standard melting enthalpy of water (334 J/g) [33]. For some groups, the prolonged immersion time and/or 37 °C temperature flattened the heat–flow curve, and no discernible transition region or peak was observed. Thermal characteristics could not be extracted for these groups, and ND (not detectable) is indicated in the tables. For instance, the absence of cold crystallization peaks within most samples immersed for 7 days at 37 °C prevented the application of the same criteria that were imposed on the other samples that possessed all of the peaks required for the calculation.

### 2.4. Water Uptake Measurement

The water uptake of samples from all groups was determined by immersing samples (three per group) in PBS at 37 °C or room temperature (RT) at different time points. The weight of the samples was measured before immersion, at 1, 2, 3, 4, and 5 days, and then weekly until water saturation was reached. The water content of the composite samples was determined gravimetrically by using Equation (2), where *M_s_* is the mass of a soaked sample and *M_d_* is the mass of a dry sample.

(2) $$Water\;uptake\%=\frac{\left(M_{s}-M_{d}\right)}{M_{d}}\times 100$$

Finally, all samples (three per group), after reaching water saturation, were mechanically tested in compression, following the protocol described in Section 2.7.

### 2.5. Dynamic Mechanical Analysis

During dynamic mechanical analysis (DMA), the elastic strain recovery of sample strips (three samples per group) with dimensions of 5 × 0.1 mm was assessed for all groups. Strips were mounted on the tensile clamp arrangement with a grip-to-grip distance of approximately 8.5 mm and subjected to 25% strain, followed by a 2 min recovery period, on a DMA 850 device (TA Instruments, New Castle, DE, USA) connected to an air chiller system (ACS3). Samples were tested under three conditions: after immersion in 37 °C PBS for 30 min, dry at 37 °C, and dry at RT. Samples at 37 °C were equilibrated at the target temperature, then extended to the target strain; the load was removed, and recovery was recorded after a 2 min period. All recovery percentages were calculated using the following equation:

(3) $$Recovery\;\left(\%\right)=\frac{L_{t}-L_{s}}{L_{s}}\times 100$$

where *L_t_* is the sample length at the time of measurement, and *L_s_* is the sample length after applying the desired strain (here, 25%).

In another series of tests, the temperature-dependent evolution of the storage moduli of samples from all groups was determined in the −90 –120 °C range. Samples underwent cycles of loading–unloading at 25% strain at 1 Hz and a heating rate of 2.5 °C min^−1^. *T_g_* was then determined by the onset method; i.e., the temperature at which a sudden drop in the storage modulus occurred was automatically recorded.

### 2.6. Gel Permeation Chromatography

Number average molecular weight (*M_n_*) and weight average molecular weight (*M_w_*) were determined by gel permeation chromatography (GPC) in dichloromethane (DCM). Samples (three per group) were dissolved in DCM and filtered to remove β-TCP where required. GPC was performed on a high-performance liquid chromatography system (Alliance e2695 Separations Module with Waters 2414 Refractive Index Detector, Waters Corporation, Milford, MA, USA). A four-column series setup was used with Waters Styragel Columns (Milford, MA, USA). Three injections per sample, each at 25 µL, were performed. Polystyrene standards were used for calibration.

### 2.7. Mechanical Testing and Recovery Rate

To evaluate the mechanical properties of LAC, γ-LAC, LTCP, and γ -LTCP samples under different conditions, five samples (height of 25.4 mm and a cross-sectional diameter of 12.7 mm) per group were tested in dry conditions (room atmosphere and RT), hydrated at 25 °C, and in simulated physiological conditions (hydrated at 37 °C). Specimens were tested under uniaxial compression according to the ASTM standard for compressive properties of rigid plastics (ASTM D695-23) [30]. Samples were compressed at a rate of 1.3 mm/min until a maximal strain of 50% with a universal testing machine 68TM (Instron, Norwood, MA, USA). Samples requiring hydration were immersed 30 min before testing, and a custom-made stainless-steel bath with temperature control was used to maintain hydration and, if required, a stable testing temperature of 37 °C, as described in our previous study [5]. The stress at the end of the test (when the strain reached 50%) was recorded and is referred to as “maximum stress” in this study. The yield stress was identified as the stress at the end of the initial linear region. The compressive modulus was calculated in the initial linear region of the stress–strain curves. Finally, the energy dissipated per unit volume by the samples was calculated from the area under the stress–strain curves in J/m^3^.

After mechanical testing, the samples from all groups were maintained under the same conditions as used during testing. To evaluate the effect of the environment on the recovery rate, the height of the samples was measured with a Vernier caliper at the following time points: 1 min, 5 min, 30 min, 1 h, 3 h, 6 h, 24 h, and 7 days. All recovery percentages were calculated using Equation (3), with *L_s_* denoting the sample length after a 50% strain.

## 3. Results and Discussion

### 3.1. Structural Characterization

The GPC results showed a clear reduction in both *M_n_* and *M_w_*, as shown in Table 2. *M_n_*, the average molecular weight by number, decreased by 53% after irradiation of polymeric samples and by 33% after irradiation of composite samples. Smaller reductions were observed in the average molecular weight (44% for LAC versus γ-LAC samples and 29% for LTCP versus γ-LTCP samples). These decreases in molecular weight were consistent with data from the literature on PLA for similar irradiation doses [34]. They can be explained by random chain scission, a mechanism well studied in PLA and other biomedical polymers, during which γ-irradiation breaks liable bonds in the polymeric chains [5,14,15,21,26,27,29,35,36,37]. Regarding PLA, a similar decrease in *M_w_* (41%) was reported by Babanalbandi et al. [15] after irradiating PLA with a 30 kGy dose. However, they observed only a 27% decrease in *M_n_*. Nugroho et al. [35] also noted that *M_n_* decreased by only 24% after irradiating PLA at 30 kGy. These differences could be attributed to the different composition of the copolymer used in this study. The lower molecular weights observed in LTCP samples compared to LAC samples are likely due to thermal degradation during compounding of the filaments used for 3D printing. However, the presence of particles probably limited chain scission. Ceramics are highly resistant to irradiation-induced damage and could have attenuated the effect of irradiation via shielding, as already observed with calcium cements embedded in polymers [38]. Finally, after irradiation, the PDI was higher in both the γ-LAC and γ-LTCP groups, indicating a broader distribution of chain sizes due to random chain scission.

The degree of crystallinity, calculated from DSC analysis, is reported in Table 3 for each group in different conditions. Composite samples exhibited higher crystallinity than polymeric samples, as previously observed in our study [5]. For instance, under dry conditions, LTCP samples exhibited a crystallinity of 4.3%, whereas that of LAC samples was 1.1%. Indeed, ceramic particles increase the crystallinity of the polymer by restricting chain mobility and acting as preferential sites for heterogeneous nucleation, which results in the growth of crystalline regions at the polymer–ceramic interface [39,40]. Furthermore, the lower molecular weight of the LTCP and γ-LTCP samples (Table 2) may have contributed to the lower crystalline content. The higher crystallinity observed in LTCP and γ-LTCP, compared to LAC and γ-LAC samples, could not be attributed to the inherent crystallinity of the β-TCP particles, given that DSC was operated at a temperature range much lower than the phase transition temperature of β-TCP, which occurs between 1125 and 1180 °C.

A higher degree of crystallinity was also observed in samples immersed in water. For instance, at 25 °C, the crystallinity of LAC samples increased to 2.8% after 30 min, 3.1% after 1 day, and 11.8% after 7 days. This result contradicts previous observations in which water molecules diffused into the polymeric matrix and dissolved crystallites [41]. However, this process is non-systematic and depends on the polymer, water content, particle type, and temperature [42,43,44]. Instead, water plasticization could have facilitated crystallite growth by increasing chain mobility in the amorphous domains. With greater mobility, polymeric chains can reorient and form hydrogen bonds, ultimately leading to expanded or new crystalline regions, as observed in PLA [45,46], poly-L-lactic acid (PLLA) [43], or other polymers [47,48,49].

Finally, γ-irradiated samples were more crystalline. γ-LAC samples exhibited a crystallinity of 23.9% under dry conditions and 27.5–37.0% in the presence of water. This increase in PLA crystallinity after irradiation is well documented in the literature [34,36,37]. Irradiation-induced chain scission reduces the polymer’s molecular weight and produces shorter segments with higher mobility, which can then reorganize into new crystallites [50,51,52]. For instance, Milicevic et al. [37] reported that the crystallinity of annealed PLLA increased from 41% to 57% after a 25 kGy irradiation dose. Interestingly, they showed that the crystallinity was maximal after a 50 kGy dose and decreased for higher doses. Zaidi et al. [34] observed that the crystallinity of PLA increased from 37% to 51% after a 30 kGy dose, and the crystallinity was also maximal at 50 kGy. γ-LTCP samples exhibited lower crystallinity (16.8% under dry conditions and 14.5–30.1% under hydrated conditions). This could be attributed to the reduced chain mobility of the polymeric chains around ceramic particles. Although our data was incomplete and we could not fully investigate the combined effect of irradiation and ceramic particles, other studies on PLA have reported that irradiation-induced crystallization was more pronounced in the presence of ceramic fillers. For instance, Dadbin et al. [53] reported that 30 vol.% hydroxyapatite–PLA composites had 12.70% crystallinity after γ-irradiation at a 30 kGy dose, while non-irradiated samples exhibited a 3.10% crystallinity.

### 3.2. Thermal Characterization

**Figure 1:**
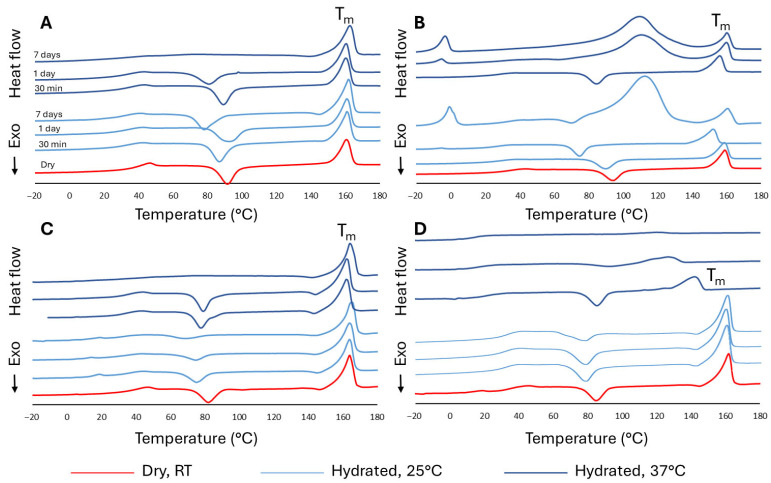
Heat flow graphs corresponding to (**A**) LAC, (**B**) LTCP, (**C**) γ-LAC, and (**D**) γ-LTCP samples. Analyses were conducted under dry conditions at RT, and after immersion in PBS at 25 °C or 37 °C for 30 min, 1 day, and 7 days.

Figure 1 displays the heat flow curves for LAC, LTCP, γ-LAC, and γ-LTCP. LAC and LTCP samples under dry conditions returned similar values for *T_m_* and *T_cc_*. However, the composite displayed a reduction in ∆*H_m_* and ∆*H_cc_* from 20.41 to 12.83 J/g and 21.0 to 14.6 J/g, respectively, because of the presence of thermally inactive β-TCP particles within the composite. Upon hydration, the *T_cc_* of both LAC and LTCP samples typically shifted to lower temperatures than in the dry samples. This shift is often associated with water plasticization [54]. The plasticization of Lactoprene™ was investigated in our previous study [5], and in addition to the thermal properties, the effect on the mechanical properties is discussed in Section 3.5. Here, the increased chain mobility of the hydrated samples during the heating ramp facilitated the development of a greater crystalline content within the matrix. The cold-crystallization enthalpy (Table S1) generally decreased with immersion time, particularly at 37 °C, and the *T_cc_* peak was often completely abolished after 7 days of immersion. Additionally, in the LTCP group (Figure 1B), a new, very broad endothermic peak was observed around 100 °C. This phenomenon could be explained by the evaporation of water absorbed earlier by the samples [55]. For samples with extensive water uptake, the endothermic evaporation process may be sufficiently strong to mask the exothermic cold crystallization event, or cold crystallization may not occur in highly hydrated samples due to excessive chain mobility in the amorphous phase. Alternatively, the polymer may have undergone hydrolytic degradation during immersion, resulting in lower-molecular-weight chains that formed new crystalline regions. The melting of these new crystallites may result in broad, large endothermic peaks in the DSC trace [56]. Similarly, another peak observed around 0 °C (melting temperature of water) indicated the presence of unbound, freezable water. From the area under the melting-temperature peak of water, the fraction of freezable water in the samples could be calculated (4.2% at both 25 °C and 37 °C after 7 days of immersion). In contrast to LCTP samples, LAC samples showed no peaks at 0 °C or 100 °C. For both LAC and LTCP samples hydrated at 37 °C, the cold-crystallization peak disappeared for immersion times of 1 day or longer, likely due to water evaporation.

Thermograms of γ-LAC and γ-LTCP samples are reported in Figure 1C,D. γ-LAC samples showed a lower *T_cc_* (81 °C) than that of LAC (92 °C). In addition, ∆*H_cc_* was lower (15.8 J/g) for γ-LAC samples compared to LAC samples (20 J/g). The reduction in *T_cc_* indicates that crystallization occurred at a lower temperature, and that the polymeric chains required less thermal energy to begin reorganizing into crystal domains. This could be attributed to increased chain mobility in the amorphous regions, resulting from irradiation-induced chain scission, as evidenced by the lower molecular weight of the irradiated samples. In addition, the reduction in ∆*H_cc_* indicates that less crystallization occurred during heating. Crystallization was therefore kinetically easier but thermodynamically less extensive during reheating.

In hydrated conditions at 25 °C, the *T_cc_* and ∆*H_cc_* of γ-LAC samples decreased at all three time points and was more pronounced after 7 days, indicating that water plasticization enhanced chain mobility, and that the presence of crystalline regions reduced ∆*H_cc_*. LAC and γ-LAC samples in hydrated conditions at 37 °C showed similar thermograms for each time point. The thermogram for γ-LTCP samples in dry conditions (Figure 1D) indicates a reduction in *T_cc_* to 84.9 °C compared to LTCP samples under dry conditions (*T_cc_* of 93.4, Figure 1B). Irradiation-induced chain scission could have facilitated chain mobility, thereby reducing the energy barrier for crystallization.

**Figure 2:**
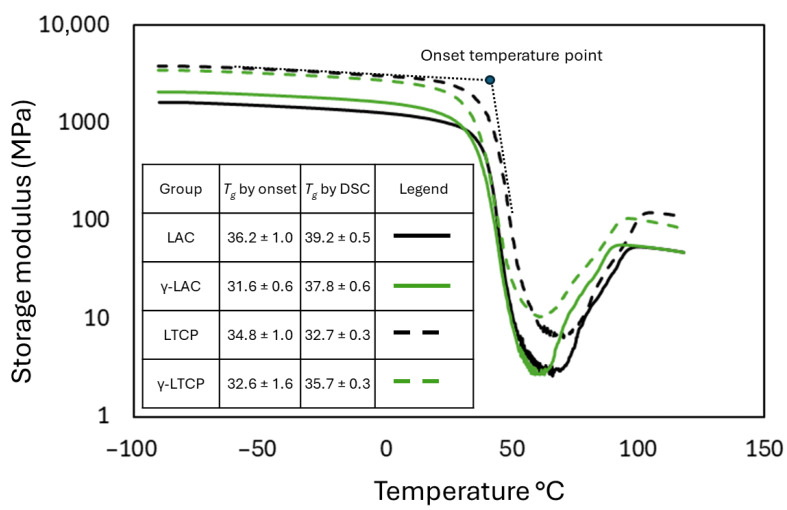
Storage modulus (*E’*) as function of temperature was measured by DMA for all groups at a heating rate of 2.5 °C·min^−1^. *T_g_* was detected automatically at the onset of the sudden decrease in the storage modulus. The comparison with DSC calculations indicates a shift of a few degrees, but the same trend. Table displays average values and standard deviations.

Table 4 presents the calculated *T_g_* values for LAC, LTCP, γ-LAC, and γ-LTCP samples obtained from DSC analysis. The composite material possessed a lower *T_g_* than the pure polymer under all comparative conditions. For hydrated samples, a reduction in *T_g_* was observed for both irradiated and non-irradiated LAC and LTCP at 25 °C and 37 °C, consistent with DMA-derived *T_g_* calculations (Figure 2) and with our previous study on Lactoprene [5]. A reduction in *T_g_* upon hydration and plasticization of a polymer is widely reported in the literature [8,57,58,59], including for PLA and PLA-derived polymers [60,61]. Indeed, the increase in chain mobility, rearrangement of the hydrogen-bond network, and formation of cavities in the polymer due to water increase the occurrence of various chain conformations [60,62]. *T_g_* can then decrease by 10–20 °C per 1 wt% increase in water content [58]. Interestingly, PLA and PLGA have a *T_g_* that decreases to below body temperature after hydration [59], underscoring the need to investigate these polymers under simulated physiological conditions.

Under dry conditions, the *T_g_* of LAC samples reduced from 39.2 °C to 37.8 °C compared to γ-LAC. The irradiation-induced structural reorganization of the polymeric network, including a lower molecular weight (as reported in Table 2), shifted the glass–rubber transition to lower temperatures, likely due to the increased segmental mobility of the shorter polymer chains in the amorphous regions.

Because *T_g_* denotes a temperature range, over which the material transitions to a rubbery state rather than a precise temperature, its value depends strongly on the calculation method. For instance, the onset method applied to the temperature evolution of the storage modulus, as shown in Figure 2, yields *T_g_* values at the onset of the transition region. With DSC, *T_g_* is identified at the midpoint of step changes in the heat capacity of the samples, as evidenced in the heat flow curve, which occurs slightly further into the transition region. The *T_g_* values calculated from DSC were therefore higher than those derived from DMA. Despite these differences, the same trend was observed with DMA calculations, with an 8–12 °C *T_g_* depression after immersion of non-irradiated samples. Irradiation apparently limited the depression of *T_g_* for polymeric samples (likely due to increased crystallinity) but increased the depression of *T_g_* at 37 °C, likely due to enhanced chain mobility, resulting in a combined effect of higher temperature and lower molecular weight.

### 3.3. Water Uptake

**Figure 3:**
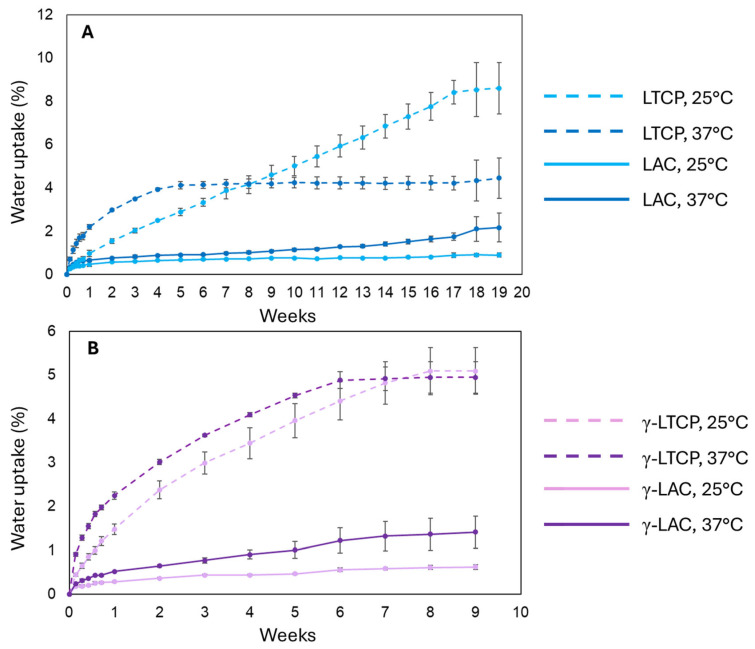
Water uptake (average and standard deviation) of samples from (**A**) LAC, LTCP groups, and (**B**) γ-LAC and γ-LTCP groups, immersed in PBS at 25 °C or 37 °C.

Figure 3 shows the water absorption profiles of irradiated and non-irradiated samples immersed in PBS at 25 °C or 37 °C. As shown in Figure 3A, composite LTCP samples absorbed water faster and to a greater extent than pure polymer LAC samples. At 25 °C, LTCP samples reached 8.5% water content, while LAC samples reached 0.9%. This pattern is common in composites with ceramic particles, as the particles promote water infiltration through ceramic–polymer interfaces [63,64]. At 37 °C, the difference was less pronounced, with LTCP samples reaching 4.5% water content and LAC samples 2.2%. This temperature dependence aligns with our previous results for non-irradiated samples, which were attributed to higher crystallinity [5]. Indeed, amorphous regions better facilitate water penetration compared with crystalline, ordered regions. Interestingly, changes in the crystalline structure during immersion could explain the temperature dependence of water uptake in LTCP samples. The rate of water absorption in LTCP samples at 37 °C was higher than at 25 °C until 8 weeks. Subsequently, samples at 37 °C plateaued, whereas samples at 25 °C continued to absorb water until 17 weeks. Although changes in the crystallinity of LTCP samples could not be measured over time, LAC samples showed lower initial crystallinity at 37 °C than at 25 °C (2.2% versus 2.8%), which could have promoted water uptake at the higher temperature. The degree of crystallinity was 18.1% at 37 °C after 24 h, compared with 3.1% at 25 °C; in the long term, this could have reduced water uptake at 37 °C.

Figure 3B shows the water-uptake profiles of γ-LAC and γ-LTCP samples at 25 °C and 37 °C. These irradiated specimens exhibited a slower water-uptake rate and a lower equilibrium water-uptake level than non-irradiated samples. Equilibrium was reached at approximately 7 days in both groups. The equilibrium water-uptake levels were 0.6% and 1.4% for γ-LAC at 25 °C and 37 °C, respectively, and 5.1% and 4.9% for γ-LTCP at 25 °C and 37 °C, respectively. A reduction in composite water uptake upon γ-irradiation has been reported previously [65], including at doses as low as 5 kGy [66]. This effect can be explained by γ-irradiation’s ability to increase the crystalline content within the polymer matrix, thereby hindering water penetration and reducing the amorphous fraction available for water to reside [14,65]. As observed for the non-irradiated samples, irradiated composite specimens exhibited higher water uptake than irradiated pure polymer specimens, owing to the presence of ceramic fillers, as previously described. Finally, the higher water uptake of γ-LAC samples at 37 °C than at 25 °C could originate from faster molecular kinetics at the elevated temperature or from lower crystallinity (23.5% after 24 h at 37 °C versus 36.5% after 24 h at 25 °C).

### 3.4. Mechanical Properties

#### 3.4.1. Effect of γ-Irradiation

**Figure 4:**
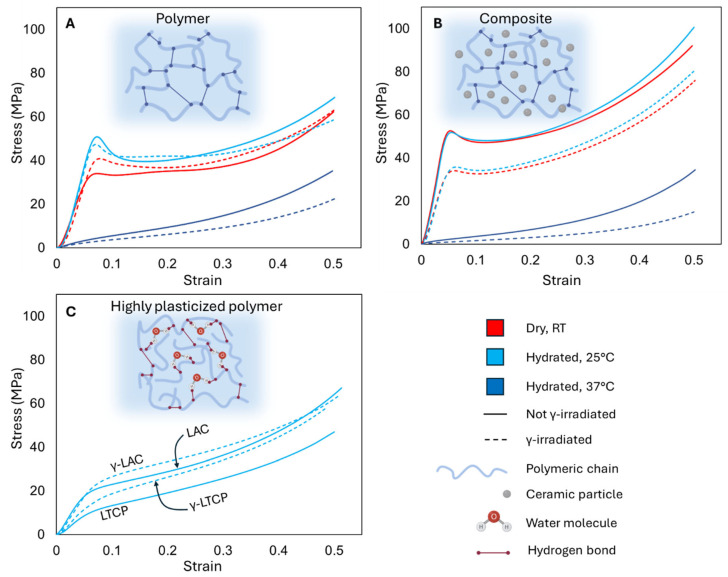
Compressive stress–strain curves for dry samples at RT, hydrated samples at 30 min at 25 °C, and hydrated samples at 30 min at 37 °C from the (**A**) LAC and γ-LAC groups and the (**B**) LTCP and γ-LTCP groups. (**C**) Compressive stress–strain curves for samples from all groups after reaching water saturation at 25 °C.

**Figure 5:**
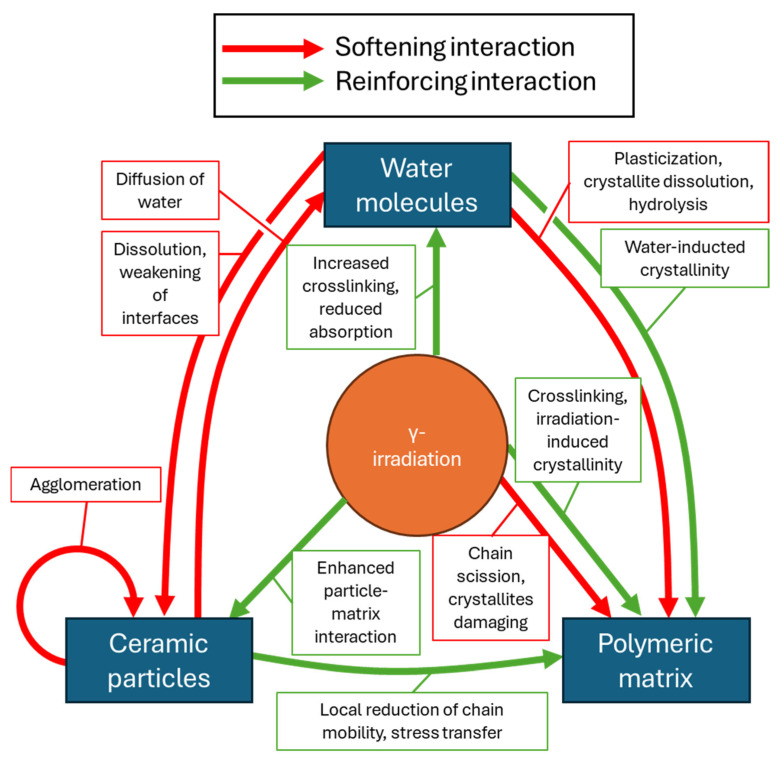
Summary of the water–ceramic–polymer interactions reported in the literature, and multiple roles of γ-irradiation that were identified in this study. Each interaction can have a reinforcing or softening effect on the mechanical properties, which explains the current challenge in designing ceramic–polymer composites for SGBR that exhibit ideal mechanical properties under physiological conditions after sterilization.

γ-irradiated and non-irradiated LAC and LTCP samples were tested under dry conditions and hydrated for 30 min at 25 °C or hydrated for 30 min at 37 °C; their stress–strain curves are shown in Figure 4A and Figure 4B, respectively. In Figure 4C, the stress–strain curves of LAC, LCTP, γ-LAC, and γ-LTCP samples tested after reaching water saturation at 25 °C are displayed. A summary of the mechanical properties is listed in Table 5. Under dry conditions, LAC and γ-LAC samples exhibited a yield stress of 26.7 MPa and 41.3 MPa and a compressive modulus of 630.7 MPa and 861.0 MPa, respectively, thereby demonstrating stiffer mechanical properties than the non-irradiated plain polymer. In the case of aliphatic polyesters, the family of biodegradable polymers used for biomedical applications, the presence of oxygen in their backbone is responsible for their high sensitivity to ionizing radiation [20,25]. Chain scission occurs primarily at ester linkages and at tertiary carbon sites [15]. An increase in yield strength was also observed by Cottam et al. [21] in PCL at a similar irradiation dose, and stiffening is commonly observed in γ-irradiated polymers [67]. This suggests that irradiation-induced crosslinking of the polymeric chains could have occurred in the samples tested in this study. In addition, the increase in crystallinity upon irradiation, as shown in Table 3, reduces chain mobility and flexibility [68]. A reinforcing effect of the crystallites can also be observed, along with plastic deformation and even brittleness [69]. Indeed, dry γ-LAC samples exhibited a crystallinity of 23.9%, whereas dry LAC samples exhibited 1.1%. However, the maximum stress and dissipated energy of LAC and γ-LAC were similar. For instance, the dissipated energy reached 18.4 MJ/m^3^ for LAC samples and 19.2 for MJ/m^3^ for γ-LAC samples. In hydrated conditions at 25 °C, the increase in the compressive modulus between LAC and γ-LAC samples was less pronounced (806.3 MPa versus 867.9 MPa) and yield strength and dissipated energy were similar, while maximum stress was larger for LAC samples, with 71.4 MPa versus 58.0 MPa for γ-LAC. The unpredictability of the mechanical properties of hydrated γ-LAC and LAC samples could arise from complex interactions among irradiation-induced structural changes, polymer content, and water content. For instance, LAC and γ-LAC exhibited different degrees of crystallinity (23.9% and 27.5%) and molecular weights (253,460 g/mol and 142,462 g/mol), as determined by DSC and GPC. γ-irradiation usually results in crystallites with a smaller size [70] that reduce the reinforcing effect and modify the interactions with water molecules. Altogether, these parameters could have induced a different sensitivity to the presence of water molecules and plasticization. These interactions are further discussed at the end of the section and in Figure 5.

#### 3.4.2. Effect of Hydration

At 25 °C, all mechanical properties of hydrated LAC samples were superior to those of dry LAC samples. Plasticization, by rearranging the polymer network and increasing polymer chain mobility, typically reduces mechanical properties and increases ductility. In the present work, the higher crystallinity in the hydrated LAC samples relative to the dry LAC samples (2.8% versus 1.1%) could have counterbalanced the plasticization effect. Surprisingly, these results contradict our previous observations, in which the same material exhibited lower mechanical properties after hydration [5]. This difference could originate from the porosity of the samples used in the previous study (10%), which facilitated water penetration into the polymer’s interior and may have accelerated plasticization. In contrast, the samples in the present study were fabricated with 0% porosity; therefore, plasticization could have been limited to the outer regions of the samples, as water penetration throughout the molecular network could take weeks. An increase in mechanical properties due to hydration at 25 °C was not observed for γ-LAC samples, despite their higher crystallinity, as evidenced by the DSC results (23. 9% for dry γ-LAC samples, 27.5% for γ-LAC samples hydrated at 25 °C). This can be explained by the lower molecular weight of this group, as indicated by the GPC results in Table 2. Because the polymeric chains in hydrated γ-LAC samples are shorter after chain scission, they may be more susceptible to water penetration and plasticization, which could counterbalance the reinforcing effect of higher crystallinity. In addition, irradiation-induced chain scission also promotes the formation of carbonyl groups (C=O) that reduce the size of the crystallites [71], as observed in PLA [34,37], for which the reinforcing effect is less efficient. For instance, Aouat et al. [36] reported that PLA crystallinity increased with irradiation dose (0–30 kGy), whereas its mechanical properties progressively decreased due to the formation of smaller crystallites. 

#### 3.4.3. Effects of Water Saturation

Samples tested after reaching water saturation, including non-irradiated samples after 19 weeks of immersion and irradiated samples after 9 weeks, exhibited worse mechanical properties due to extensive plasticization. All samples immersed at 25 °C exhibited hyperelastic behavior (Figure 4C), which is further discussed in Section 3.4.4. Samples immersed at 37 °C could not be tested due to advanced hydrolytic degradation.

The mechanical properties of γ-LAC (or γ-LTCP samples) were slightly superior to those of LAC (or LTCP samples), as observed previously in samples immersed for only 30 min. However, this difference can be attributed to immersion time rather than irradiation: non-irradiated samples were immersed twice as long (19 weeks versus 9 weeks) to reach saturation, which may have increased degradation. Interestingly, the presence of particles was not advantageous after a long immersion, as LTCP’s (or γ-LTCP’s) mechanical properties were lower than those of LAC (or γ-LAC). For instance, the moduli of LTCP and γ-LTCP samples were 219.8 MPa and 278.3 MPa, respectively, while the moduli of LAC and γ-LAC samples were 432.2 MPa and 391.5 MPa, respectively. For long immersion times, the increased water diffusion caused by the presence of ceramic particles likely counterbalanced the effect of matrix reinforcement. Water-associated mechanisms, such as crystallite dissolution [41,72], weakening of ceramic–matrix interfaces [73,74], or hydrolytic degradation [72,75,76], could occur at higher rates, degrading the properties of the samples. Ceramic particles likely promoted water diffusion in composite samples, thereby accelerating their degradation. Therefore, the samples still exhibited relatively high mechanical properties after long-term immersion at 25 °C, which were compatible with SGBR application. However, the increased hydrolytic degradation observed at 37 °C could be problematic for clinical translation. Biodegradability is an advantageous property in the context of SGBR; however, it must match the remodeling rate of bone, which is around 6 weeks [77]. Further in vitro and in vivo investigations on the biodegradation rate of the material must be conducted to evaluate if the material maintains its mechanical properties for a period of 6 weeks. Additional studies on the role of temperature on the accelerated degradation must also be conducted to identify strategies to control degradation in physiological conditions.

#### 3.4.4. Effect of Temperature

When the temperature under hydrated conditions increased from 25 °C to 37 °C, above the *T_g_* of the hydrated materials, all mechanical properties of both LAC and γ-LAC samples decreased dramatically, and the mechanical response shifted from a semi-crystalline profile to an S-shaped curve typical of hyperelasticity. This mechanical profile reflects a transition among three regimes that is typical of rubber elasticity: the “knee region,” stiffer due to the outward push of internal pressure from mobile polymeric chains as deformation begins; the quasi-linear region, where molecular chains are progressively stretched, producing a linear response to strain; and the strain-hardening region, where the molecular network approaches its maximum extension and the chains can no longer support further stretching [78,79]. The increased mobility of the polymeric chains, due to the combined effects of a temperature above *T_g_* and hydration, likely favored the emergence of these three regimes [78] and conferred hyperelasticity to the material. At 37 °C, the compressive moduli of LAC and γ-LAC samples decreased to 55.0 MPa (11-fold decrease) and 43.7 MPa (19-fold decrease), respectively. In hyperelasticity, polymeric chains can slide over one another during deformation, thereby reducing resistance to deformation and lowering the Young’s or compressive moduli [80]. The effect was amplified in the γ-LAC samples, likely because the polymer’s molecular weight decreased after irradiation, thereby reducing entanglement and friction during chain sliding.

#### 3.4.5. Effect of Ceramic Particles

LTCP specimens exhibited superior mechanical properties, including compressive modulus and yield strength, in the dry state under hydrated conditions at 25 °C and 37 °C, compared with the LAC group, due to reinforcement from β-TCP fillers within the polymer matrix. This reinforcement is commonly observed in ceramic-reinforced polymers [39,81,82,83,84], and in this study it counteracted the decrease in molecular weight measured by GPC in composite samples. Ceramic particles promote crystal growth by acting as nucleation sites, as discussed in Section 3.1. In addition, a matrix with higher crystallinity can bond more strongly with the particles, thereby improving stress transfer [85]. The increase in crystallinity, therefore, enhanced the mechanical properties. However, at high ceramic contents (40 wt% and above), studies have reported that moduli could still be improved, but strength was dramatically reduced [8,86,87,88,89,90]. The loss of strength is due to the tendency of particles to agglomerate, driven by their high surface energy, into a state of stable equilibrium, which creates undesirable defects in the structure [91,92]. Here, the higher strength observed in the composite with 40 wt% ceramic can be attributed to optimal dispersion of ceramic particles during fabrication, as evidenced by our previous study [5] and by other investigations of composites with high contents of uniformly distributed ceramic particles [93,94]. 

#### 3.4.6. Combined Effects of Irradiation and Ceramic Particles

All mechanical properties of γ-LTCP samples tested in dry or hydrated conditions were lower compared to LTCP samples in the same conditions. This result contradicts prior studies reporting that γ-irradiation enhances filler–polymer interactions, thereby improving mechanical properties [95,96,97]. However, some studies investigated the combined effects of γ-irradiation and a high ceramic content on aliphatic polyesters and identified conditions under which irradiation did not improve mechanical properties. For instance, Suljovrujic et al. [98,99] remarked that PLLA–hydroxyapatite nanocomposites with 80% ceramic content had lower mechanical properties after a 50 kGy dose of irradiation. Similarly, Dadbin et al. [29] observed a significant decrease in the tensile strength and elongation at break in PLA–hydroxyapatite nanocomposites with 50 vol% ceramic after irradiation at 30 kGy. Interestingly, the material in the present study, fabricated from Lactoprene (74% lactide units) with 40 wt% ceramic and irradiated at 25 kGy, was similar to that tested by Dadbin et al., who reported results comparable to ours (a decrease in tensile strength from 35 MPa to 20 MPa after irradiation). Dadbin et al. attributed this change to PLA’s tendency to degrade via chain scission at this irradiation dose and to additional interactions between radiation and the inorganic phase. Indeed, the presence of the particles affects the polymeric structure in their vicinity, as the thermal motion of the molecular chains is restricted by the ceramic particles [100,101]. This mechanism is amplified in composites with high ceramic contents, thereby locally affecting crystallinity [39,40] or increasing stress concentration [102]. This mechanism may have altered the irradiation-induced crosslinking process, leaving only chain scission in the polymer chains. Further investigations using infrared spectroscopy or solid-state and time-domain nuclear magnetic resonance spectroscopy are needed to assess the crosslink density, chain mobility, and interfacial strength between the ceramic particles and the matrix, thereby elucidating the presence of such mechanisms.

#### 3.4.7. Combined Effects of Irradiation, Ceramic Particles, and Physiological Conditions

Interestingly, the reinforcing effect of hydration at 25 °C, described earlier for the LAC and γ-LAC groups, was also observed for the LTCP and γ-LTCP groups, as all mechanical properties were slightly higher under these conditions than in the dry state for the same groups. However, a dramatic reduction in all mechanical properties of the LTCP and γ-LTCP groups was observed in physiological conditions compared with other conditions, as already observed for the LAC and γ-LAC groups.

γ-LTCP samples exhibited better mechanical properties than γ-LAC samples under dry and hydrated conditions at 25 °C, but not when hydrated at 37 °C. In physiological conditions, the compressive modulus was 26.0 MPa for γ-LTCP versus 43.7 MPa for γ-LAC, and the maximum stress was 17.2 MPa for γ-LTCP versus 22.0 MPa for γ-LAC. This can be explained by a combination of factors that adversely affected the polymeric structure of γ-LTCP samples: irradiation reduced the polymer’s molecular weight; ceramic particles may have facilitated water penetration, thereby amplifying the plasticization effect [63,64] and weakening the polymer–ceramic bond [103]; and the temperature was above *T_g_*. Overall, chain mobility was exacerbated, and the mechanical properties were the lowest observed across all groups and conditions, underscoring the challenges associated with fabricating biodegradable polymer–ceramic composites for SGBR that remain stable under simulated physiological conditions. These observations could have important consequences in the field of SGBR, where the load-bearing property of bone implants is essential to improve the recovery of the patient. Bone implants must be capable of supporting loads not only to provide mobility and quality of life to the patient but also to transfer mechanical stresses to the bone–implant interface, which promotes bone growth and osteointegration [104,105]. Here, we demonstrated that, although γ-irradiation can improve the mechanical properties of dry samples, the opposite is observed in physiological conditions. Given that 25 kGy is the minimal dose recommended to ensure appropriate sterilization of biomedical implants, and that higher doses are more likely to induce additional structural damage such as chain scission, our results suggest rethinking the sterilization process in a way that minimizes the loss of mechanical properties. Indeed, the present study conducted the sterilization of samples before immersion in water, to mimic a clinical scenario where sterile samples are implanted in a patient. However, studies on silicone foams have evidenced that the absorbed moisture in the polymer could reflect the structural changes induced by γ-irradiation [106]. Future investigations must therefore be conducted to determine if immersion of biodegradable polymers in water, until the appropriate water uptake is reached, before sterilization could be an effective way to deliver implants with improved load-bearing capability for SGBR applications.

#### 3.4.8. Molecular-Scale Interactions in Irradiated Composites in Physiological Conditions

Overall, the macroscale differences between the sample groups can be explained only through in-depth investigations of the interactions among polymeric chains, ceramic particles, and water molecules at the molecular scale. Irradiation produces competing effects: an increase in crystallinity and crosslinking on the one hand, and chain scission and a decrease in molecular weight on the other. Ceramic particles usually increase the mechanical properties, but they also facilitate the absorption of water, which then weakens the ceramic–polymer interface via dissolution of the particles and plasticizes the matrix. Water can enhance mechanical properties via water-induced crystallinity but can also plasticize the matrix, along with temperature, when it reaches the glass transition temperature. The difficulty in predicting the mechanical behavior of ceramic-reinforced polymers, therefore, arises from the complexity of these interactions, which is summarized in Figure 5. Further investigation at the molecular scale with infrared spectroscopy, nuclear magnetic resonance spectroscopy, non-linear optical spectroscopy, or molecular dynamics simulations could provide fundamental information on the state of water, on the type of bonding between water, polymer, and particles, or (at a lower scale) on the vibrational dynamics of hydrogen bonds [8]. Such investigations could deepen our understanding of this complex interplay and support predicting the properties observed at the macroscale.

### 3.5. Recovery

**Figure 6:**
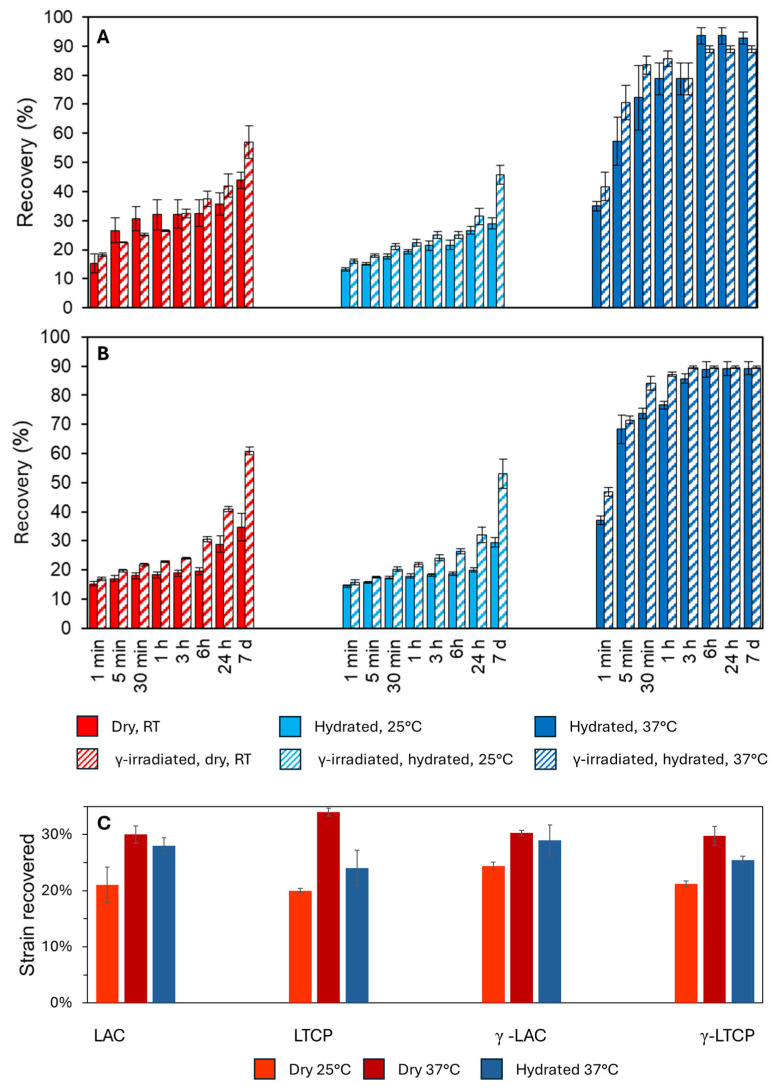
(**A**) Recovery behavior of LAC and γ-LAC samples and (**B**) LTCP and γ-LTCP samples after compressive test under dry conditions at RT, hydrated at 25 °C, and hydrated at 37 °C conditions. The recovery percentage of the cylindrical samples was monitored after compression to 50% of their initial length, and averaged. Error bars indicate standard deviations. (**C**) Short-term (2 min) recovery behavior of LAC, LTCP γ-LAC and γ-LTCP samples, under dry at 25 °C, dry at 37 °C, and hydrated at 37 °C conditions. The recovery percentage of the sample strips was monitored after applying a 25% tensile strain and averaged. Error bars indicate standard deviations.

During mechanical testing, cylindrical specimens were maintained under the same conditions as during the tests, and the recovery rate of each group was monitored. Figure 6 shows the recovery behavior over time for LAC, γ-LAC, LTCP, and γ-LTCP samples. Samples tested in a hydrated condition at 25 °C exhibited the lowest recovery (29.0% for LAC samples after 7 days), whereas those tested after hydration at 37 °C had the highest recovery rate (92.8% for LAC samples after 7 days). Recovery requires polymeric chains to slide past one another, driven by crosslinks that do not break during the compression test. This process is facilitated by the reduction in hydrogen-bonding strength at temperatures above *T_g_*, as observed in our previous study [5]. Samples therefore exhibited a remarkable recovery capability characteristic of hyperelasticity. Water plasticization also promotes recovery by reducing the number of hydrogen bonds, thereby accelerating recovery from elastic deformation [60,107]. However, in this study, water-induced crystallinity may account for the lower-than-expected recovery of samples hydrated at 25 °C. The rapid recovery of composite materials for SGBR applications can be advantageous in certain contexts, such as minimally invasive surgery, where a pre-compressed implant is inserted, expands as body temperature is reached, and exerts appropriate stress on the surrounding bone tissue, which stimulates remodeling [108,109]. 

The effect of irradiation was not noticeable for polymeric or composite samples hydrated at 37 °C; recovery was already maximal, with approximately 90% achieved after 6 h in both cases. However, irradiation improved elastic recovery in dry and hydrated conditions at 25 °C. For instance, after 7 days, the recovery of γ-LAC and γ-LTCP samples hydrated at 25 °C reached 45.7% and 53.1%, respectively, whereas that of LAC and LTCP samples reached 29.0% and 29.5%, respectively. This could be attributed to the shorter molecular chains obtained after irradiation, which were more mobile. In addition, the denser network of crosslinks induced by irradiation likely contributed to the restoration of the polymeric chains to their original arrangement. A similar observation applies to samples in dry conditions, since the recovery of γ-LAC and γ-LTCP samples reached respectively 57.0% and 60.8%, while the recovery of LAC and LTCP samples was only 43.9% and 40.8%, respectively.

The short-term elastic recovery of LAC, γ-LAC, LTCP, and γ-LTCP samples was measured with DMA, through a different testing protocol (25% strain was applied in tension and released). After two minutes, the recovered strain was measured and is reported in Figure 6C. In this analysis, samples were tested in three conditions: dry at 25 °C, dry at 37 °C, and hydrated at 37 °C. The highest 2 min recovery was observed in all samples under dry conditions at 37 °C, whereas the lowest was observed under dry conditions at 25 °C. A higher temperature, above *T_g_*, therefore increased recovery because of enhanced chain mobility, whereas hydration had a damping effect on elastic recovery. Hydration-induced crystallinity, as evidenced by the DSC results, or the reduction in entanglements upon hydration, could have slightly counterbalanced the effects of the higher temperature on recovery. The effect of hydration was more visible for LTCP and γ-LTCP samples. Indeed, β-TCP particles, being hydrophilic, could have facilitated water infiltration and enhanced their effect. Finally, irradiation did not significantly affect short-term recovery, as also observed for time points of 3 h or less in Figure 6.

Future research should target molecular dynamics at atomic and sub-femtosecond timescales. Recent progress in characterization methods, such as X-ray free-electron lasers, soft X-ray second-harmonic generation, vacuum ultraviolet free-electron lasers, and ultrafast two-dimensional infrared spectroscopy [110,111,112,113], allows for the investigation of electron interactions with high sensitivity. This enables the detection of phenomena like changes in electronic topology, providing opportunities for observing quantum materials [114,115] and possibly revealing the underlying mechanisms discussed in this work.

## 4. Conclusions

The present work investigated the effects of hydration, temperature, and γ-irradiation, a common technique for surgical sterilization, on the structural, thermal, and mechanical properties of 3D-printed scaffolds fabricated from a medical-grade composite. The results revealed that at an irradiation dose of 25 kGy, chain scission, induced crystallinity, or crosslinking were observed, thereby affecting the thermal and mechanical properties. Water and temperature affected the mobility of the polymeric chains and may have facilitated the reorganization of hydrogen-bond networks or the assembly of highly ordered regions. The complex interplay among particles, water molecules, and polymeric chains, influenced by temperature and irradiation, remains incompletely understood but is essential for understanding the macroscale physicochemical properties observed.

## Figures and Tables

**Table 1:** Composition and characteristics of the groups of samples tested in this study.

Group Name	Geometry and Composition	Irradiation
LAC	Lactoprene^®^, bulk-like cylindrical 12.7 × 25.4 mm	No
γ-LAC		γ-irradiated
LTCP	Lactoprene^®^/β-TCP (60:40), bulk-like cylindrical 12.7 × 25.4 mm	No
γ-LTCP		γ-irradiated

**Table 2:** Number average molecular weight (*M_n_*), weight average molecular weight (*M_w_*), and *M_w_*/*M_n_* ratio (polydispersity index, PDI) obtained from GPC, for all groups, with standard deviations.

	LAC	γ-LAC	LTCP	γ-LTCP
M_n_	156,830 ± 2082	73,705 ± 314	115,505 ± 4041	77,018 ± 3749
M_w_ (g/mol)	253,406 ± 491	142,462 ± 907	205,690 ± 1465	146,140 ± 707
PDI	1.61 ± 0.02	1.93 ± 0.01	1.79 ± 0.05	1.90 ± 0.08

**Table 3:** Degree of crystallinity (%), *X_c_*, measured from DSC analysis, for all groups and different conditions of immersion (average and standard deviation). ND: Not detectable.

Condition	Group			
	LAC	LTCP	γ-LAC	γ-LTCP
Dry	1.1 ± 0.5	4.3 ± 0.2	23.9 ± 2.5	16.8 ± 0.5
25 °C–30 min	2.8 ± 0.1	7.1 ± 0.8	27.5 ± 1.4	14.5 ± 3.0
25 °C–1 d	3.1 ± 1.2	7.7 ± 0.3	36.5 ± 2.4	15.8 ± 1.6
25 °C–7 d	11.8 ± 1.5	ND	37.0 ± 2.0	30.1 ± 2.7
37 °C–30 min	2.2 ± 0.5	6.4 ± 0.5	25.3 ± 0.9	1.9 ± 0.3
37 °C–1 d	18.1 ± 0.1	ND	23.5 ± 1.9	3.95 ± 1.5
37 °C–7 d	ND	ND	ND	ND

**Table 4:** Temperature of glass transition, *T_g_*, in °C, measured from DSC analysis, for all groups, and different conditions of immersion (average and standard deviation).

Condition	Group			
	LAC	LTCP	γ-LAC	γ-LTCP
Dry	39.2 ± 0.5	32.7 ± 0.3	37.8 ± 0.6	35.7 ± 0.3
25 °C–30 min	29.2 ± 0.6	24.3 ± 1.8	34.4 ± 1.2	28.4 ± 2.2
25 °C–1 d	33.8 ± 0.9	26.2 ± 0.4	34.3 ± 0.7	30.1 ± 1.0
25 °C–7 d	29.0 ± 0.8	ND	33.6 ± 0.6	31.4 ± 0.2
37 °C–30 min	36.4 ± 0.6	26.3 ± 0.6	33.5 ± 1.0	23.7 ± 0.9
37 °C–1 d	31.1 ± 0.6	ND	33.4 ± 0.7	16.4 ± 0.3
25 °C–7 d	27.4 ± 0.7	ND	34.4 ± 2.3	14.6 ± 0.7

**Table 5:** Summary of the mechanical properties for samples from all groups and testing conditions, including compressive modulus, maximum and yield stress, and dissipated energy (average and standard deviation). Yield stress could not be calculated for samples exhibiting hyperelastic behavior. ND: Not detectable.

Testing Conditions	Group	Compressive Modulus (MPa)	Max Stress (MPa)	Yield Stress (MPa)	Energy Dissipated (MJ/m^3^)
Dry, RT	LAC	630.7 ± 40.6	60.5 ± 3.1	26.7 ± 3.5	18.4 ± 0.7
	γ-LAC	861.0 ± 36.1	61.3 ± 2.3	41.3 ± 0.6	19.2 ± 0.9
	LTCP	1142.7 ± 93.9	89.2 ± 2.9	41.6 ± 2.0	27.1 ± 1.0
	γ-LTCP	795.2 ± 43.4	74.8 ± 1.4	32.6 ± 1.3	21.3 ± 0.3
Wet, 25 °C	LAC	806.3 ± 45.0	71.4 ± 4.7	43.2 ± 1.0	22.8 ± 1.7
	γ-LAC	867.9 ± 75.1	58.0 ± 6.7	43.4 ± 3.0	20.9 ± 0.4
	LTCP	1177.2 ± 69.5	95.1 ± 5.9	43.7 ± 2.0	29.1 ± 1.1
	γ-LTCP	965.6 ± 65.4	83.7 ± 2.7	38.5 ± 2.2	23.9 ± 1.1
Water saturated, 25 °C	LAC	432.2 ± 22.6	66.1 ± 1.6	ND	18.2 ± 0.4
	γ-LAC	391.5 ± 43.0	61.9 ± 2.8	ND	18.5 ± 0.8
	LTCP	219.8 ± 10.3	46.9 ± 0.1	ND	10.7 ± 0.6
	γ-LTCP	278.3 ± 2.2	55.8 ± 1.5	ND	14.2 ± 0.3
Wet, 37 °C	LAC	55.0 ± 11.2	24.1 ± 3.9	ND	5.4 ± 0.9
	γ-LAC	43.7 ± 8.8	22.0 ± 5.8	ND	4.6 ± 1.1
	LTCP	77.3 ± 9.3	33.2 ± 1.8	ND	5.7 ± 0.4
	γ-LTCP	26.0 ± 5.7	17.2 ± 3.2	ND	2.9 ± 0.5

## Data Availability

The raw data supporting the conclusions of this article will be made available by the authors on request.

## Supplementary Materials

The following supporting information can be downloaded at: https://www.mdpi.com/article/10.3390/polym18070817/s1, Table S1: Supplemental results extracted from DSC analysis.

## Abbreviations

The following abbreviations are used in this manuscript:

β-TCP	β-tricalcium phosphate
ASTM	American Society for Testing and Materials
CE	Conformité Européenne
DCM	Dichloromethane
DMA	Dynamic mechanical analysis
DSC	Differential scanning calorimetry
FDA	Food and Drug Administration
GPC	Gel permeation chromatography
ND	Not detectable
PBS	Phosphate-buffered saline
PCL	Polycaprolactone
PDI	Polydispersity index
PDLLA	Poly-d,l-lactic acid
PLA	Poly-lactic acid
PLGA	Poly(lactic-co-glycolic acid)
PLLA	Poly-L-Lactic Acid
PTMC	Poly-trimethylene carbonate
RT	Room temperature
SGBR	Scaffold-guided bone regeneration
STL	Stereolithography
TPEs	Thermoplastic elastomers
