# Supplementary material for: Effect of Gamma Irradiation and Simulated Physiological Conditions on the Physicochemical Properties of a 3D-Printed βTCP Composite
Source: Polymers (Basel). 2026 Mar 27;18(7):817. doi: 10.3390/polym18070817 (PMC13074754; doi:10.3390/polym18070817)
Supplement: Supplementary file 1 [file polymers-18-00817-s001.zip › polymers-4189846-supplementary.pdf]

Supplementary material

Table S1: Supplemental results extracted from DSC analysis: T_{cc} (cold crystallization temperature), ΔH_{cc} (cold crystallization enthalpy), T_m (melting temperature), ΔH_m (melting enthalpy) and freezable water content, for all groups under different conditions of immersion (average and standard deviation). ND: Non detectable.

**Group and conditions**	T_{cc} (°C)	ΔH_{cc} (J/g)	T_m (°C)	ΔH_m (J/g)	**Freezable water content (%)**
LAC – Dry	92.2 ± 0.3	-20.4 ± 0.6	161.0 ± 0.3	21.0 ± 0.4	ND
LAC – 25°C – 30 min	85.3 ± 1.5	-21.0 ± 0.7	161.1 ± 0.7	24.1 ± 1.0	ND
LAC – 25°C – 1 d	90.7 ± 2.3	-23.2 ± 0.6	161.4 ± 0.8	23.2 ± 0.7	ND
LAC – 25°C – 7 d	80.0 ± 3.0	-21.6 ± 0.9	162.2 ± 0.8	27.9 ± 2.5	ND
LAC – 37°C – 30 min	90.6 ± 1.2	-19.2 ± 2.4	160.4 ± 0.1	21.0 ± 0.7	ND
LAC – 37°C – 1 d	78.6 ± 3.4	-10.7 ± 2.7	160.8 ± 0.4	24.5 ± 1.5	ND
LAC – 37°C – 7 d	NC	NC	162.2 ± 1.0	27.7 ± 1.3	ND
LTCP – Dry	93.4 ± 0.3	-12.8 ± 0.1	158.9 ± 0.5	14.6 ± 0.2	ND
LTCP – 25°C – 30 min	90.1 ± 1.0	-14.0 ± 0.3	159.0 ± 0.5	13.6 ± 0.05	ND
LTCP – 25°C – 1 d	75.6 ± 0.9	-11.0 ± 0.6	156.4 ± 3.0	13.8 ± 0.2	0.2 ± 0.02
LTCP – 25°C – 7 d	ND	ND	160.0 ± 0.5	13.5 ± 0.8	4.2 ± 0.3

LTCP – 37°C – 30 min	84.6 ± 0.9	-12.3 ± 0.6	156.7 ± 1.2	13.4 ± 0.1	ND
LTCP – 37°C – 1 d	ND	ND	160.3 ± 0.3	15.5 ± 0.8	1.0 ± 0.1
LTCP – 37°C – 7 d	ND	ND	159.7 ± 0.2	13.5 ± 1.0	4.2 ± 0.5
γ-LAC – Dry	81.1 ± 1.5	-15.8 ± 1.0	163 ± 0.3	32.2 ± 0.7	ND
γ-LAC – 25°C – 30 min	72.6 ± 2.0	-14.0 ± 0.6	163.8 ± 0.6	33.0 ± 0.4	ND
γ-LAC – 25°C – 1 d	73.6 ± 1.2	-8.0 ± 1.9	164.2 ± 0.3	33.1 ± 0.3	ND
γ-LAC – 25°C – 7 d	68.3 ± 0.2	- 8.2 ± 0.4	164.7 ± 0.3	33.6 ± 1.0	ND
γ-LAC – 37°C – 30 min	80.0 ± 0.02	-19.2 ± 0.02	162.3 ± 0.1	36.6 ± 0.6	ND
γ-LAC – 37°C – 1 d	79.9 ± 1.0	-20.7 ± 0.1	162.4 ± 0.3	36.8 ± 1.5	ND
γ-LAC – 37°C – 7 d	ND	ND	164.1 ± 0.2	36.0 ± 0.1	ND
γ-LTCP – Dry	84.9 ± 0.2	-11.3 ± 0.1	161.6 ± 0.1	18.3 ± 0.2	ND
γ-LTCP – 25°C – 30 min	78.8 ± 0.2	-13.4 ± 1.3	160.4 ± 0.3	19.4 ± 0.1	ND
γ-LTCP – 25°C – 1 d	79.8 ± 0.9	-13.1 ± 0.4	160.3 ± 0.5	19.3 ± 0.3	ND
γ-LTCP – 25°C – 7 d	75.1 ± 4.5	-6.7 ± 0.3	160.9 ± 0.1	19.2 ± 0.8	ND
γ-LTCP – 37°C – 30 min	85.7 ± 0.6	-11.5 ± 0.1	142.0 ± 0.5	12.3 ± 0.1	ND

γ-LTCP – 37°C – 1 d	93.4 ± 1.0	-5.0 ± 0.5	127.0 ± 0.2	6.6 ± 0.1	ND
γ-LTCP – 37°C – 7 d	ND	ND	122.0 ± 1.6	1.6 ± 0.5	ND
